# Hepatic Schistosomiasis: An Overlooked Diagnosis in Non-endemic Regions

**DOI:** 10.7759/cureus.77945

**Published:** 2025-01-24

**Authors:** Mathew Vadukoot Lazar, George S Zacharia, Amit H Shejal, Hadik A Patel, Priya J Mathew, Baiju F Puthenkote, Rajesh Paraswani, Ipsita Panda, Jessymol Joseph

**Affiliations:** 1 Gastroenterology, Lifecare Hospital, Abu Dhabi, ARE; 2 Gastroenterology and Hepatology, Malankara Orthodox Syrian Church (MOSC) Medical College Hospital, Cochin, IND; 3 Gastrointestinal Surgery, Lifecare Hospital, Abu Dhabi, ARE; 4 Dentistry, Lifecare Hospital, Abu Dhabi, ARE; 5 Internal Medicine, Lifecare Hospital, Abu Dhabi, ARE; 6 Radiodiagnosis, Lifecare Hospital, Abu Dhabi, ARE; 7 Pathology and Laboratory Medicine, Burjeel Medical City, Abu Dhabi, ARE; 8 Nursing, Lifecare Hospital, Abu Dhabi, ARE

**Keywords:** anti-smooth muscle antibodies, hepatic fibrosis, liver biopsy, portal hypertension, schistosomiasis

## Abstract

Schistosomiasis, caused by trematodes of the genus *Schistosoma*, is one of the most frequent parasitic infections worldwide. *Schistosoma mansoni* and *Schistosoma japonicum* are responsible for most hepatosplenic schistosomiasis cases, infections culminating in granulomatous inflammation, portal fibrosis, and portal hypertension. Definitive diagnosis requires detecting schistosome eggs in stool or biopsy samples, as anti-schistosomal antibodies cannot differentiate active from past infections. Treatment involves a single dose of praziquantel (40 mg/kg) administered orally. Here, we report a young male patient from an endemic region, presenting as incidentally detected mild but persistent transaminitis, who, upon evaluation, was diagnosed with hepatic schistosomiasis with portal fibrosis and portal hypertension. Although he had a low titer positive serology for anti-smooth muscle antibodies (ASMA), the diagnosis of autoimmune hepatitis (AIH) was excluded with a liver biopsy. The case underscores the importance of considering schistosomiasis in unexplained liver disease among patients from or traveled to endemic regions, emphasizing the need for a high clinical suspicion to optimize timely diagnosis and intervention.

## Introduction

Schistosomiasis is one of the most frequent human parasitic infections and the most frequent cause of portal hypertension worldwide, affecting around 200 million people globally. However, it has garnered less attention globally than its counterparts owing to its highly restricted geographical preponderance, mainly in North and Sub-Saharan Africa. Hepatic schistosomiasis is most frequently caused by *Schistosoma mansoni* (*S. mansoni*) and *Schistosoma japonicum* (*S. japonicum*) [1]. The schistosomal ova in the intrahepatic portal venous system incites inflammatory response, granulomatous reaction, and fibrosis but typically has limited effect on hepatocytes or the hepatic lobular architecture, unlike in cirrhosis. The end result is portal fibrosis-related portal hypertension, which can be complicated by gastrointestinal bleeds and hypersplenism [1-3].

## Case presentation

**Figure 1:**
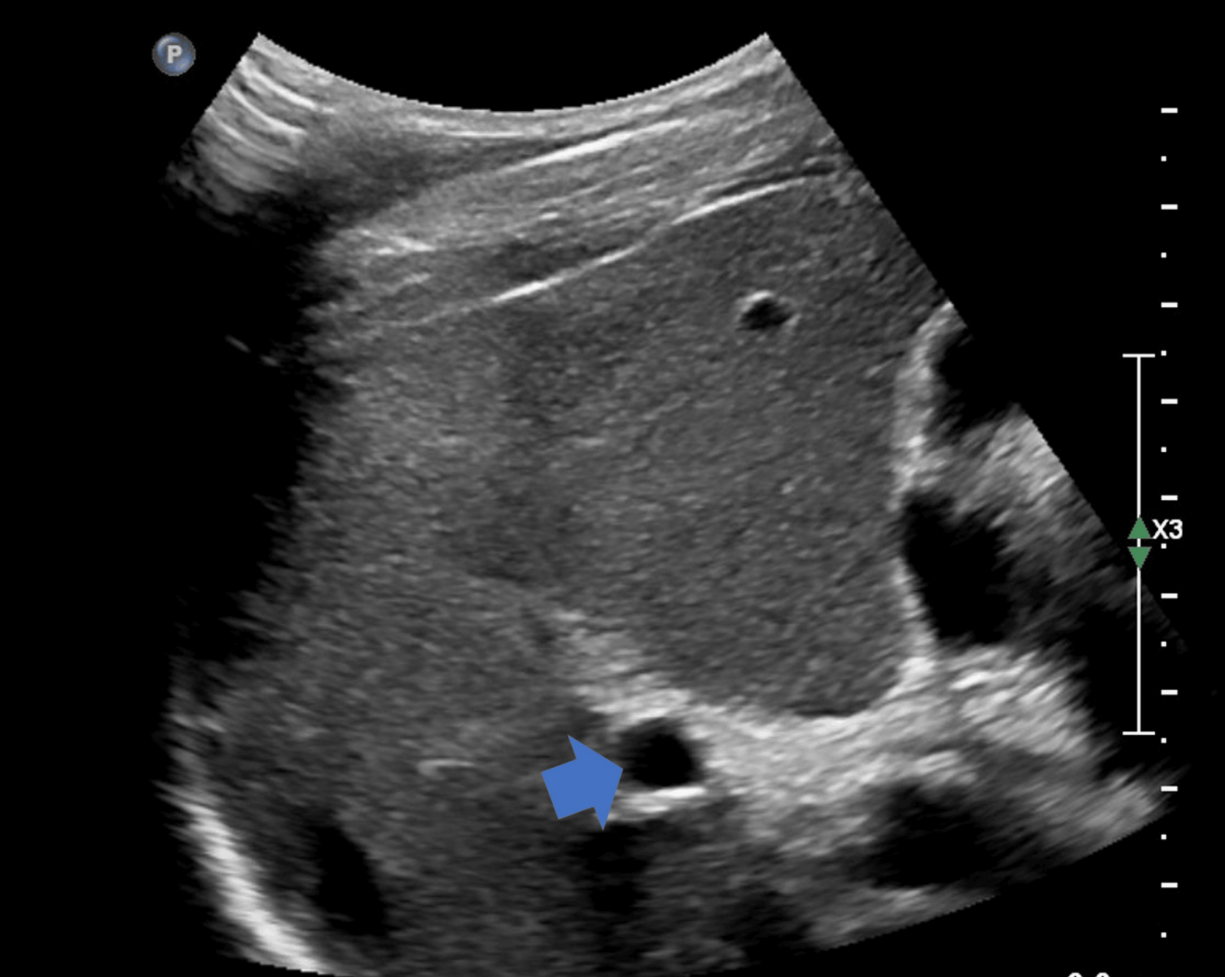
Ultrasound of the liver revealing a prominent portal vein (blue arrow) and heterogeneous liver echoes

**Figure 2:**
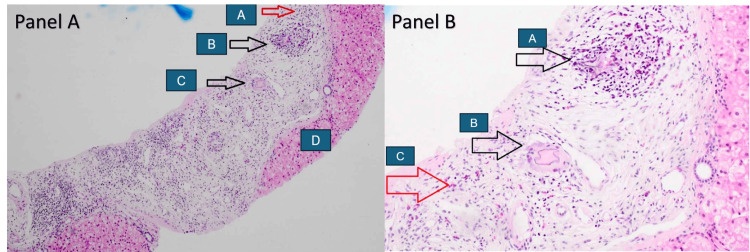
Photomicrographs of liver biopsy specimens Panel A: Low magnification images reveal an expanded portal tract displaying increased fibrosis, edema, and moderate mixed inflammation composed of predominantly eosinophils, lymphocytes, and plasma cells. (A) Collections of inflammatory cells with predominance of eosinophils, (B) nodular aggregate with multinucleated giant cell in the center surrounded by eosinophil predominant inflammatory infiltrate, (C) multinucleated giant cell with the presence of degenerate Schistosoma egg, and (D) no evidence of interface hepatitis. Panel B: Higher magnification view demonstrating (A) nodular aggregate with multinucleated giant cell in the center surrounded by eosinophil predominant inflammatory infiltrate, (B) multinucleated giant cell with the presence of degenerate eggs, and (C) increased eosinophils in the portal tract.

**Figure 3:**
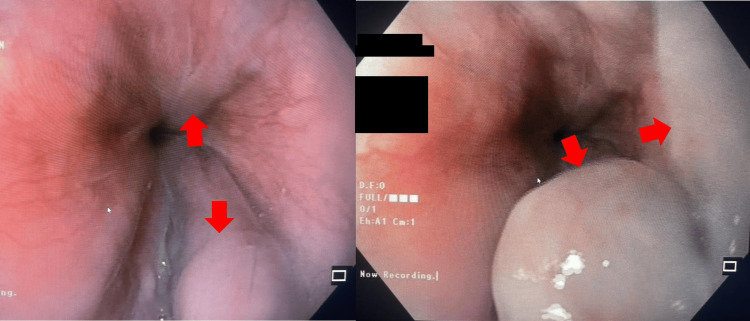
Endoscopic images revealing esophageal varices (red arrows)

A 32-year-old male patient from Uganda was referred to the gastroenterology clinic for the evaluation of persistent altered liver enzymes for the past six months. He had no apparent symptoms throughout the entire course. He denied the use of ethanol or long-term prescription or complementary medications. Family history was significant for maternal chronic liver disease, with no further details available. The hemogram was normal except for eosinophilia (14%) with an absolute eosinophil count of 960 cells/mm^3^. Routine biochemistry revealed minimal transaminitis (Table 1). Abdominal sonography revealed coarse liver echoes and a borderline enlarged spleen, consistent with chronic liver disease and portal hypertension (Figure 1). There were no hepatic mass lesions, ascites, cholelithiasis, or evidence of biliary obstruction. Viral hepatitis B surface, core antigens, and antibodies to hepatitis A, C, and E were undetectable. The antimitochondrial and antinuclear antibodies were negative; however, anti-smooth muscle antibodies (ASMA) were low positive (1:40). Immunoglobulin assay revealed an elevated serum IgE level (181 IU/mL) (normal: <100 IU/mL). Anti-schistosomal antibodies (IgG) were positive (4.4) (normal: <0.8). The stool analysis was within normal limits and did not reveal the presence of any ova or parasites. Given the dilemma between schistosomiasis and autoimmune hepatitis (AIH), the patient was recommended a liver biopsy. Following informed consent, an ultrasound-guided liver biopsy was performed, which revealed portal fibrosis and chronic inflammation composed of lymphocytes, eosinophils, and multinucleated giant cells with degenerate helminthic ova (Figure 2). At biopsy, the lobular architecture was primarily maintained, special stains were negative for storage diseases, and there were no histological features to suggest AIH. An upper gastrointestinal endoscopy revealed grade II esophageal varices with no stigmata of recent bleeding (Figure 3). A diagnosis of hepatic schistosomiasis with portal fibrosis and portal hypertension was made, and he was administered a single oral dose of praziquantel (40 mg/kg). He tolerated the medication without any adversities and was subsequently discharged. On follow-up, he continues to be asymptomatic.

**Table 1:** Summary of the laboratory workup AST: aspartate transaminase, ALT: alanine transaminase, ALP: alkaline phosphatase

Parameter	Results		Reference range
Hemoglobin (gm/dL)	Baseline	3 months follow-up	14-18
	14.6	14.7	
Leukocyte count (cells/μL)	6850	5200	4000-11000
Absolute eosinophil count (cells/μL)	960	312	50-400
Platelet count (cells/μL)	162000	194000	150000-450000
Bilirubin total/direct (mg/dL)	1.1/0.6	0.8/0.3	0.1-1.2/<0.4
AST/ALT (IU/L)	71/89	54/60	<40/<48
ALP (IU/L)	138	125	35-145
Ceruloplasmin (mg/dL)	24	-	20-35
Transferrin saturation	28%	-	<45%
Antinuclear, anti-liver kidney microsomal, antimitochondrial, anti-tissue transglutaminase, anti-neutrophil cytoplasmic antibodies	Negative	-	Negative
Anti-smooth muscle	Positive (1:40)	-	Negative

## Discussion

Schistosomiasis, or bilharziasis, is a parasitic infection caused by trematodes belonging to six discrete species: *S. japonicum*, *S. mansoni*, *Schistosoma haematobium*, *Schistosoma intercalatum*, *Schistosoma mekongi*, and *Schistosoma malayensis* [2]. It is one of the most frequent human parasitic infections, affecting around 200 million people every year worldwide [2,4]. *Schistosoma haematobium* results in urogenital schistosomiasis, while other species are responsible for gastrointestinal and hepatobiliary schistosomiasis [5]. Schistosomal hepatopathy is most frequently caused by *S. mansoni* and, to a lesser extent, by *S. japonicum* [1]. Humans and other mammals constitute the definitive host, while freshwater snails remain the intermediate hosts. The infective form, cercariae, is a free-swimming larvae found in fresh waters in the endemic regions. The cercaria gains access to humans by penetrating the skin, finds its way to the veins, and is carried by the blood flow to the liver. They mature into adult male and female flatworms in the liver, migrate to the mesenteric or vesical venous system, and generate eggs. The eggs are shed through the feces or urine; encountering fresh water allows the continuation of the life cycle. Eggs are also swept by portal blood flow, allowing them to reach the hepatic portal microcirculation. The eggs in the liver elicit an initial Th1, subsequently evolving into a Th2 immune response, resulting in eosinophilic infiltration, granuloma formation, and ultimately fibrosis [1,3]. The end result is severe portal fibrosis, also known as Symmers pipe stem fibrosis. Unlike in cirrhosis, the hepatic lobular architecture is largely preserved and devoid of regenerative nodules [1].

Hepatic schistosomiasis is regarded among the most frequent causes of portal hypertension, although Egypt, Yemen, and Algeria bear the brunt of the disease [1]. Those affected are frequently asymptomatic. Portal hypertension-related gastrointestinal bleeding or hypersplenism are the most frequent manifestations. Hepatocellular failure is uncommon, although a subset of patients might develop end-stage liver disease over a protracted course [6,7]. Concurrent infections with viral hepatitis B or C or human immunodeficiency virus have been associated with accelerated disease progression and mortality [3]. Literature from China has demonstrated an association between *S. japonicum* infection and hepatocellular cancers; however, the carcinogenic potential warrants further evidence [8]. A definitive schistosomiasis diagnosis mandates egg demonstration in stool, urine, or tissue biopsy. Serology detects anti-schistosomal antibodies; however, it is limited by its inability to differentiate between active and past infections, as it can remain positive for long periods. Circulating cathodic antigen assays in urine and polymerase chain reaction to schistosomal antigens or DNA in urine, stools, blood, or tissue are being evaluated to identify active infection. Abdominal imaging helps identify portal hypertension, splenomegaly, and hepatic changes, although nonspecific. Endoscopic evaluation allows the detection of portal hypertensive changes, including gastroesophageal varices, and achieves hemostasis in most cases of bleeding [1,3,9]. Treatment with praziquantel, a single dose of 40 mg/kg, is indicated in patients diagnosed with schistosomiasis. Monitoring treatment response is often challenging; however, the load of egg excretion may be assayed 4-6 weeks after praziquantel administration, but applicable only in egg excretors at baseline [9].

Our patient, hailing from an endemic region, presented with incidentally detected, persistent mild transaminitis. Although nonspecific, his eosinophilia pointed toward a parasitic etiology, and his serology was positive for anti-schistosomal antibodies, suggesting hepatobiliary schistosomiasis. However, the evaluation revealed an ASMA-positive status, raising concerns about simultaneous AIH or even isolated AIH, as anti-schistosomal antibodies do not differentiate active from past schistosomiasis. Hence, a liver biopsy was recommended for further confirmation of diagnosis as the treatment and prognosis vary between the two diagnoses. The liver histology was convincing for hepatic schistosomiasis and had no features suggestive of AIH. A review of published literature identified reports of autoimmune marker-positive status, antinuclear or anti-smooth muscle, or anti-parietal cell antibodies in low titers in up to 15% of hepatosplenic schistosomiasis [10]. Also, low titers of ASMA have been demonstrated in other liver diseases, autoimmune and rheumatologic diseases, and various infections [11]. The young man was confirmed to have hepatic schistosomiasis with portal fibrosis and portal hypertension, treated with a single dose of praziquantel, and is being followed up in the clinic.

## Conclusions

Schistosomiasis is a common cause of liver disease and portal hypertension. As the disease is primarily restricted to specific geographic regions, it is often overlooked as a cause of liver disease in the rest of the world. The lack of awareness about the disease, the intrinsic limitations, and the nonspecificity of diagnostic modalities make the diagnosis challenging outside the endemic regions. This report highlights the need to maintain a high index of suspicion of hepatic schistosomiasis in patients with otherwise unexplained liver disease or portal hypertension in those from or who have traveled to endemic regions.
